# Bulb of *Lilium longiflorum* Thunb Extract Fermented with *Lactobacillus acidophilus* Reduces Inflammation in a Chronic Obstructive Pulmonary Disease Model

**DOI:** 10.4014/jmb.2301.01022

**Published:** 2023-02-17

**Authors:** Ji-Eun Eom, Gun-Dong Kim, Young In Kim, Kyung min Lim, Ju Hye Song, Yiseul Kim, Hyeon-Ji Song, Dong-Uk Shin, Eun Yeong Lim, Ha-Jung Kim, Sung Hoon Kim, Deuk Sik Lee, So-Young Lee, Hee Soon Shin

**Affiliations:** 1 Food Functionality Research Division, Korea Food Research Institute (KFRI), Wanju 55365, Republic of Korea; 2 Department of Food Biotechnology, Korea University of Science and Technology (UST), Daejeon 34113, Republic of Korea; 3 Department of Food Science and Technology, Jeonbuk National University, Jeonju 54896, Republic of Korea; 4 BOTANOS, Gangneung 25451, Republic of Korea; 5 WellbeingLS, Gangneung 25451, Republic of Korea

**Keywords:** *Lilium longiflorum* Thunb bulb, *Lactobacillus acidophilus*, chronic obstructive pulmonary disease (COPD), anti-inflammation

## Abstract

Chronic obstructive pulmonary disease (COPD), one of the leading causes of death worldwide, is caused by repeated exposure to harmful matter, such as cigarette smoke. Although *Lilium longiflorum* Thunb (LLT) has anti-inflammatory effects, there is no report on the fermented LLT bulb extract regulating lung inflammation in COPD. Thus, we investigated the protective effect of LLT bulb extract fermented with *Lactobacillus acidophilus* 803 in COPD mouse models induced by cigarette smoke extract (CSE) and porcine pancreas elastase (PPE). Oral administration of the fermented product (LS803) suppressed the production of inflammatory mediators and the infiltration of immune cells involving neutrophils and macrophages, resulting in protective effects against lung damage. In addition, LS803 inhibited CSE- and LPS-induced IL-6 and IL-8 production in airway epithelial H292 cells as well as suppressed PMA-induced formation of neutrophil extracellular traps in HL-60 cells. In particular, LS803 significantly repressed the elevated IL-6 and MIP-2 production after CSE and LPS stimulation by suppressing the activity of the nuclear factor kappa-light-chain-enhancer of activated B (NFκB) in mouse peritoneal macrophages. Therefore, our results suggest that the fermented product LS803 is effective in preventing and alleviating lung inflammation.

## Introduction

Chronic obstructive pulmonary disease (COPD) is a respiratory disease characterized by irreversible airflow limitation, emphysema, inflammatory airway obstruction, and destruction of the alveolar wall [1]. It is caused by repeated exposure to cigarette smoke (CS) or harmful particulate matter and occurs mainly in smokers and people over 40 years old. It is a major cause of death worldwide and its prevalence over the years has continuously increased [2]. Although smoking is the major cause of COPD, exposure to household and air pollution, chemicals, and occupational dust is also believed to contribute to this condition [3].

During the inflammatory response, various cytokines and chemokines regulate the activation and recruitment of inflammatory cells [4]. Irritants such as CS inhaled into the respiratory tract can activate airway epithelial cells and macrophages to release several chemotactic mediators, particularly chemokines, which attract circulating neutrophils, lymphocytes, and monocytes to the lungs [5]. Macrophages and neutrophils are known to play an important role in chronic inflammation in patients with COPD [6], and an increase in macrophages and neutrophils has been observed in the airways, sputum, and bronchoalveolar lavage fluid (BALF) of patients with COPD [7, 8]. During COPD exacerbation, macrophages and neutrophils influence the progression and acceleration of tissue destruction in emphysema and acute/chronic bronchitis associated with CS [9, 10]. Macrophages exposed to CS produce interleukin (IL) 6, and this inflammatory cytokine also contributes to the pathogenesis of emphysema and COPD [11]. In addition, macrophages activated by cigarette smoke extract (CSE) release inflammatory mediators such as tumor necrosis factor a (TNF-α), C-X-C motif chemokine ligand (CXCL) 1, and C-C motif chemokine ligand 2 (CCL-2), otherwise known as monocyte chemoattractant protein 1 (MCP-1) [5]. Recruitment of neutrophils to the lungs causes local inflammation and induces irreversible cell death and tissue repair through progenitor cells [12]. Recruited neutrophils and resident alveolar macrophages increase the secretion of matrix metalloprotease 2 (MMP-2), MMP-9, and MMP-12 [5, 13]. In addition, activated neutrophils increase the formation of neutrophil extracellular traps (NETs), neutrophil elastase, and MMPs [14]. It has been reported that the activity and production of MMP-14 is increased in the airway epithelium of mice exposed to cigarette smoke [15].

The plants of the genus *Lilium*, including *L. lancifolium*, *L. pumilum*, *L. longiflorum*, *L. callosum*, and *L. brownie* are used as food or medicine in many parts of the world, particularly Asia, and grown as ornamental plants. They have also been reported to have anti-tumor [16], anti-inflammatory [17, 18], anti-oxidant [19], immune function-enhancing [20], hypoglycemic, anti-bacterial, anti-depressant, anti-fatigue, and anti-hypoxic effects. Among them, Easter lily, *Lilium longiflorum* Thunb (LLT), bulbs contain a variety of bioactive phytochemicals, including phenolics, flavonoids, carotenoids, alkaloids, and steroidal saponins, in addition to other beneficial components, such as dietary fiber, microelements, and starch [21]. Therefore, they can be used not only in cooking but also as traditional medicinal treatments with anti-tussive, anti-inflammatory, and sedative effects [22].

According to a recent study, a methanol extract of *L. lancifolium* root inhibited nitric oxide (NO), prostaglandin E2 (PGE2), IL-6, TNF-α, inducible nitric oxide (iNOS), and cyclooxygenase-2 (COX-2) in lipopolysaccharide (LPS)-induced RAW264.7 cells [17]. In addition, a water extract of *L. lancifolium* root reduced the number of macrophages and neutrophils in the BALF of CS-exposed mice and inhibited IL-6, TNF-α, IL-1bβ, and MCP-1 in the BALF and lung tissue [23]. A methanol extract of *L. brownie* var. *viridulum* bulb inhibited IL-6, TNF-α, IL-1bβ, iNOS, PGE2, COX-2, NFκB p65, and MAPK in LPS-induced RAW264.7 cells [18], confirming the anti-inflammatory effect of the lily genera, not only in the root but also in the bulb. However, there have been no studies evaluating the activity of bulb extracts from the species LLT in COPD mouse models induced by CSE and porcine pancreas elastase (PPE), and studies on anti-inflammatory activity are also lacking. In this study, the attenuating effects of an LLT bulb extract fermented with *Lactobacillus acidophilus* were investigated in a CSE- and PPE-induced COPD mouse model.

## Materials and Methods

### Plant Material and Preparation

Fresh LLT bulbs were purchased from Gangneung Lily Farming Association Corp. (Korea). Sumizyme AC cellulase was obtained from Bision Biochem Ltd. (Korea). The bulbs were washed with cold water and dried at 50°C for 12 h in an air drying oven (JW-500ED, Jinwoo Electric Co., Korea). The dried bulbs were steamed at 95°C for 1 h, followed by a 1 h incubation at 50°C, and this step was repeated two additional times. The dried bulbs were then ground using an electric grinder (Model SJC, Sung Jin Precision, Korea) and sifted through a 50-mesh sieve before 50 g of the powder was added to 1 L of distilled water in an Erlenmeyer flask. The mixture was incubated at 95°C for 3 h in a thermostatic bath with agitation, after which the pH of the mixture was adjusted to 4. Sumizyme AC (0.05 g) was then added to the mixture and incubated at 50°C for 3 h. Finally, the mixture was centrifuged at 5,000 ×*g* for 10 min at 4°C (Mega 17R, Hanil Science Industrial, Korea), after which the supernatant was collected and sterilized at 121°C for 15 min.

### Fermentation

*Lactobacillus acidophilus* 803 KCTC14740BP isolated from coffee cherry was pre-cultured at a density of 1–2 × 10^9^ CFU/ml in MRS broth (Difco, USA) at 37°C for 16 h before 1% of the pre-cultured *L. acidophilus* 803 was inoculated into the sterilized extract. The culture was then fermented at 37°C for 15 h, followed by sterilization at 121°C for 15 min. The fermented and sterilized culture (LS803) was concentrated at 60°C using a vacuum evaporator (Rotavapor R-200; Buchi, Switzerland) and freeze-dried (FDT-8612; Operon Co. Ltd., Korea). To evaluate the efficacy of LS803, the raw material before fermentation (LLT) was used as the comparison group.

### Preparation of the Cigarette Smoke Extract

The CS was extracted using a 3R4F research cigarette purchased from the University of Kentucky (USA) and a vacuum pump. The unfiltered cigarette was burned for 1 min and the generated CS was passed through 10 ml phosphate-buffered saline (PBS; Welgene, Korea) using a vacuum pump. It was then filtered through a MILLEX LCR hydrophilic polytetrafluoroethylene 0.45-μm filter (Merck-Millipore, USA), after which the optical density was measured at a wavelength of 320 nm using an Epoch Microplate Spectrophotometer (BioTek, USA). To standardize manufacturing conditions, only CS extracts with an absorbance value at 320 nm between 0.9–1.2 were used in the experiment. The CSEs were stored at –80°C until use.

### Measurement of Human IL-6 and IL-8 Levels in NCI-H292 Cells

NCI-H292 human lung epithelial cells were obtained from the Korean Cell Line Bank (Korea), and cultured in RPMI-1640 medium containing 10% fetal bovine serum (FBS) and 1% penicillin-streptomycin. RPMI-1640, FBS, and antibiotics were purchased from Welgene. The cells were seeded into 48-well cell culture plates at a density of 8 × 10^4^ cells/well and cultured for 24 h, followed by starvation in RPMI-1640 medium containing 0.2% FBS at 37°C for 24 h. The cells were then co-treated with LLT or LS803 while stimulated with 2% CSE and 100 ng/ml LPS. Post-treatment, the culture supernatants were collected and the cytokine levels were measured using the enzyme-linked immunosorbent assay (ELISA).

### Measurement of NETosis in HL-60 Cells

HL-60 human peripheral blood lymphoblast cells were obtained from the American Type Culture Collection (USA) and cultured in RPMI-1640 medium containing 10% FBS and 1% penicillin-streptomycin. The cells were seeded into 96-well black plates at a density of 1 × 10^5^ cells/well and incubated for 30 min. After incubation, the cells were co-treated with 100 nM Phorbol 12-myristate 13-acetate (PMA; Sigma Aldrich, USA) and 200 μg/ml LS803 at 37°C for 3 h with 5% CO_2_. SYTOX Green dye (Invitrogen, USA) was diluted 500-fold with Hank’s balanced salt solution (Welgene) and added to the wells, followed by incubation at 25°C for 10 min. The fluorescence intensity was analyzed at 480/530 nm using a SpectraMax i3 (Molecular Devices, USA).

### Peritoneal Macrophage Collection and Western Blot Analysis

Animal experiments were conducted in accordance with the guidelines of the Institutional Animal Care and Use Committee of the Korea Food Research Institute (approval no. KFRI-M-22035). All the mice were maintained under specific pathogen-free conditions at a temperature of 23 ± 2°C and relative humidity of 50 ± 5%, under a 12 h/12 h light/dark cycle with free access to food and water. To obtain peritoneal macrophages (PMs), 2 ml of sterile 3% Brewer Thioglycollate Medium (Sigma Aldrich) solution was injected intraperitoneally into mice and peritoneal macrophages were then collected after 3 days using an 18-G needle and syringe. The collected peritoneal macrophages were seeded into 6-well cell culture plates at a density of 3 × 10^6^ cells/well. The medium in the plates was replaced with fresh medium after 6 h and the cells were then cultured at 37°C for 24 h with 5% CO_2_. The cells were pre-treated with LLT or LS803 for 1 h before stimulation and then co-treated with LLT or LS803 while stimulating with 2% CSE and 100 ng/ml LPS, for 15 min. To extract the protein, the PMs were collected in Dulbecco’s phosphate-buffered saline and the cytoplasm and nuclei were separated using NE-PER Nuclear and Cytoplasmic Extraction Reagent (Thermo Fisher Scientific, USA). The concentrations of the extracted proteins were measured using the Bradford protein assay. Protein expression was determined by western blot analysis using a previously published method [25, 26]. The expression of nuclear factor of kappa light polypeptide gene enhancer in B-cells inhibitor, alpha (IκBα) and β-actin (Cell Signaling Technology, USA) in the cytoplasmic fraction, and nuclear factor kappa-light-chain-enhancer of activated B cell (NFκB), p65, and lamin B1 (Cell Signaling Technology) in the nuclear fraction, were measured using the ChemiDoc XRS+ (Bio-Rad, USA). The cultured supernatants were collected, and the cytokine levels were measured using ELISA.

### Animal Study

Five-week-old male BALB/C mice (20 g) were obtained from Orient Bio Inc. (Korea), acclimated for 1 week, and divided into six groups (*n* = 8): control (naive), CSE- and PPE-treated (COPD), 10 mg/kg roflumilast (ROF)-treated (positive control), 200 mg/kg LLT-treated, 100 mg/kg LS803-treated, and 200 mg/kg LS803-treated groups. For the induction of experimental COPD, the mice were intranasally treated with 20 μl PPE (1.8 U/head) on days 8, 15, and 22, and 20 μl CSE on days 8, 10, 12, 15, 17, and 19 after being anesthetized with isoflurane. From day 1 to day 22, daily treatments were administered orally with the naive group receiving PBS, while LLT and LS803 groups received LLT or LS803, respectively; diluted in PBS. ROF was administered daily from day 8 to day 22 as a positive control. The mice were euthanized on day 23 and their blood, BALF, and lung tissues were collected for further study. The left lungs were collected and excised, the lung tissue to be used for histological analysis was immediately fixed in 10% neutral buffered formalin solution, and the lung tissue for mRNA extraction was stored at -80°C until used in the experiment. The blood samples from each mouse were analyzed using an automatic blood cell automatic analyzer (ADVIAÒ 2120i Blood Cell Automatic Analyzer, Siemens, Germany).

### Immune Cell Counting and Diff-Quik Staining of the BALF

The total cell count in the BALF was measured using an ADAM-MC automatic cell counter (NanoEntek, Korea). The BALF was centrifuged at 300 ×*g* for 5 min to separate the cell pellets and supernatants, and the collected supernatant was used to measure cytokine levels in the BALF by ELISA. The cell pellets were resuspended in a volume of PBS equal to the volume of BALF collected from the mice, and the cell suspension was attached to the coated slides by centrifugation at 1,000 ×*g* for 10 min using a Shandon Cytospin 4 Cytocentrifuge (Thermo Fisher Scientific). Cells on the slides were stained using the Diff-Quik staining reagent (SYSMEX, Japan), according to the manufacturer’s instructions, and the number of neutrophils and macrophages was estimated using an Olympus BX40 microscope (Olympus, Japan).

### Measurement of Cytokines and Chemokines in the BALF

The levels of cytokines and chemokines in the BALF were measured using a Q-Plex Assay Kit (Quansys Biosciences, USA). The calibration standard solution and BALF samples were diluted at a ratio of 1:2 with a sample diluent, added to Q-Plex Array 96-well plates and shaken at 25°C for 1 h. After washing, streptavidin-horseradish peroxidase was added and the plate was shaken at 25°C for 15 min. The substrate solution was then added to the plate and the results were visualized using a Q-View Imager LS (Quansys Biosciences). The concentrations of cytokines and chemokines were analyzed using the Q-View software (Quansys Biosciences).

### RNA Extraction from the Lung Tissues and Real-Time Quantitative Polymerase Chain Reaction (qPCR)

To extract mRNA from the lung tissues, QIAzol Lysis Reagent and sterile beads were added to the individual lung samples collected from each group. The tissues were homogenized four times for 20 s at 20 Hz, using a TissueLyser II (Qiagen, USA), and then extracted using an RNeasy Mini Kit (Qiagen), according to the manufacturer's instructions. The extracted RNA was quantified using an Epoch Microplate Spectrophotometer. cDNA was synthesized in a C1000 Thermal Cycler (Bio-Rad) at 45°C for 1 h, after which the qPCR was performed on a CFX Connect Real-Time PCR System (Bio-Rad). The sequences of the primers used were as follows: IL-6 forward, 5¢-TAGTCCTTCCTACCCCAATTTCC-3¢ and reverse, 5¢-TTGGTCCTTAGCCACTCCTTC-3¢; MMP-12 forward, 5¢-CTGCTCCCATGAATGACAGTG-3¢ and reverse, 5¢-AGTTGCTTCTAGCCCAAAGAAC-3¢; MMP-14 forward, 5¢-GGACTGAGATCAAGGCCAAT-3¢ and reverse, 5¢-GCCCACCTTAGGGGTGTAAT-3¢; CXCL-1 forward, 5¢-AGACTCCAGCCACACTCCAA-3¢ and reverse, 5¢-TGACAGCGCAGCTCATTG-3¢; CCL-2 forward, 5¢-TTAAAAACCTGGATCGGAACCAA-3¢ and reverse, 5¢-GCATTAGCTTCAGATTTACGGGT-3¢; CCL-4 forward, 5¢-TTCCTGCTGTTTCTCTTACACCT-3¢ and reverse, 5¢-CTGTCTGCCTCTTTTGGTCAG-3¢; GAPDH, used as housekeeping gene: forward, 5¢-AGGTCGGTGTGAACGGATTTG-3¢ and reverse, 5¢-TGTAGACCATGTAGTTGAGGTCA-3¢.

### Histological Analysis of the Lung Tissues

Histological analysis of lung tissues was performed by hematoxylin and eosin (H&E) staining using a previously published method [25]. The excised lung tissues from each group were immediately immersed in 10% buffered formalin solution, fixed, and after dehydration embedded in paraffin. The lung tissues were sectioned and stained with hematoxylin and eosin before observation and image capture using an Olympus BX40 microscope.

### Blood Collection and Cell Population Analysis

The numbers of red blood cells, white blood cells, platelets, and reticulocytes in the blood were measured and the composition ratio of lymphocytes, monocytes, neutrophils, and eosinophils constituting the white blood cells was analyzed.

### Measurement of Immunoglobulin and Cytokine Levels

Immunoglobulin and cytokine levels in the serum or culture supernatant were measured using commercially available ELISA kits for human IL-6 (BD Biosciences, USA), human IL-8 (BD Biosciences), IgA Mouse Uncoated ELISA Kit with Plates (Thermo Fisher Scientific), and IgG (Total) Mouse Uncoated ELISA Kit with Plates (Thermo Fisher Scientific), according to the manufacturer’s instructions.

### Statistics

All data were analyzed using GraphPad Prism version 9.0 (GraphPad Software, USA). Multiple groups were statistically compared using one-way analysis of variance (ANOVA) followed by a least significant difference test. The in vitro and in vivo data have been expressed as mean ± SD. A *p*-value of < 0.05 was considered statistically significant (*p < 0.05, **p < 0.01, and ***p < 0.001).

## Results

### Effect of LS803 on Inflammatory Cell Infiltration, Cytokines, and Chemokines in the BALF

**Fig. 1:**
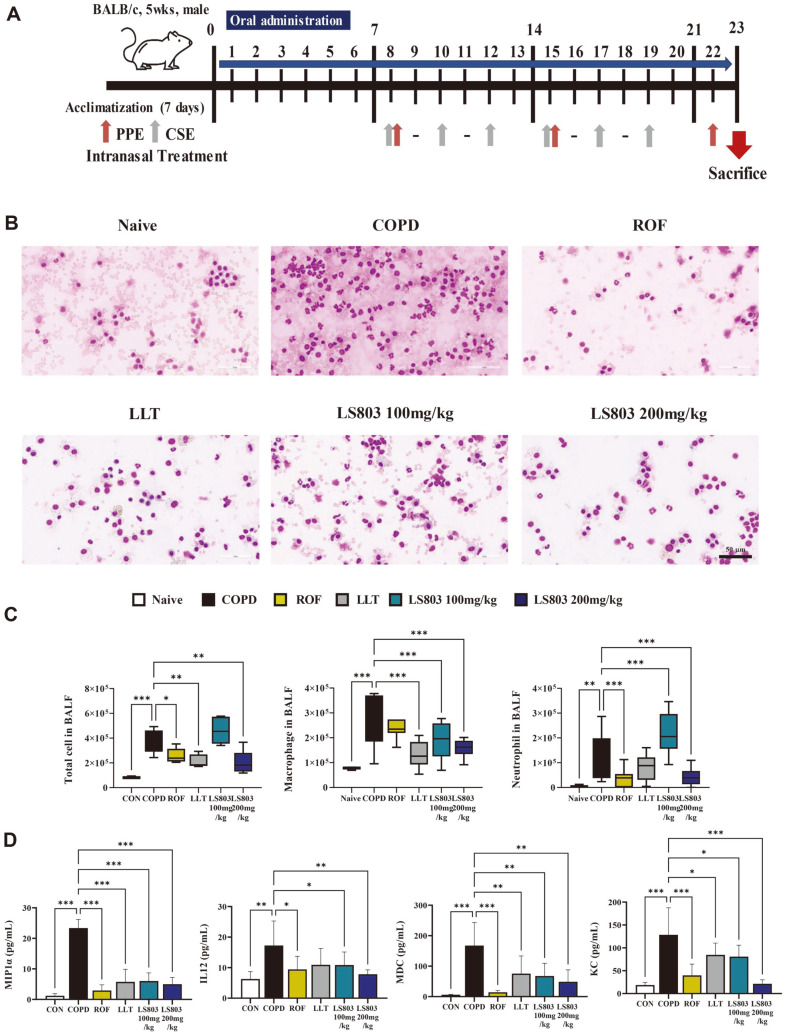
Effect of LS803 on the BALF from the COPD model. (**A**) Schematic of the CSE- and PPE-induced COPD model timeline. (**B**) The inflammatory cell infiltration was measured using Diff-Quik staining methods (scale bar: 50 μm). (**C**) Total cell, macrophage, and neutrophil numbers in the BALF. The number of immune cells was estimated using microscopy images. (**D**) The levels of cytokine and chemokines were measured using a Q-Plex Assay Kit (*n* = 5). The data were analyzed using one-way analysis of variance (ANOVA) and presented as mean ± SD (*p < 0.05, **p < 0.01, and ***p < 0.001). LLT, bulbs of *Lilium longiflorum* Thunb (the raw material before fermentation); LS803, fermented material.

COPD was induced using CSE and PPE based on a previously established COPD mouse model [24-26]. Oral administration of LLT and LS803 was initiated 7 days before CSE and PPE induction to identify the prophylactic effects. ROF was administered as a positive control from day 8 to maintain COPD exacerbation (Fig. 1A). The total number of cells increased in the BALF of mice treated with CSE and PPE compared to the naïve group. Diff-Quik staining of BALF cells and microscopic observation confirmed an increase in the number of macrophages and neutrophils (Fig. 1B). In contrast, treatment with LLT and LS803 decreased the number of inflammatory cells in the mice. In the BALF, a decrease in the total cell number was not observed in the 100 mg/kg LS803-treated group owing to the neutrophil count. However, a decrease was observed when a higher concentration of LS803 (200 mg/kg) was used, thereby confirming a concentration-dependent effect (Fig. 1C). Measuring the levels of cytokines and chemokines in the BALF revealed an increase in the levels of macrophage inflammatory protein 1 alpha, IL-12, macrophage-derived chemokine, and keratinocyte-derived chemokine in the COPD group; this was attenuated upon administration of LLT or LS803. In addition, there was a greater reduction when 200 mg/kg LS803 was orally administered compared to that seen in the LLT treatment group (Fig. 1D).

### Effect of LS803 on Immunoglobulin Levels and Hematological Parameters in the Mice

**Fig. 2:**
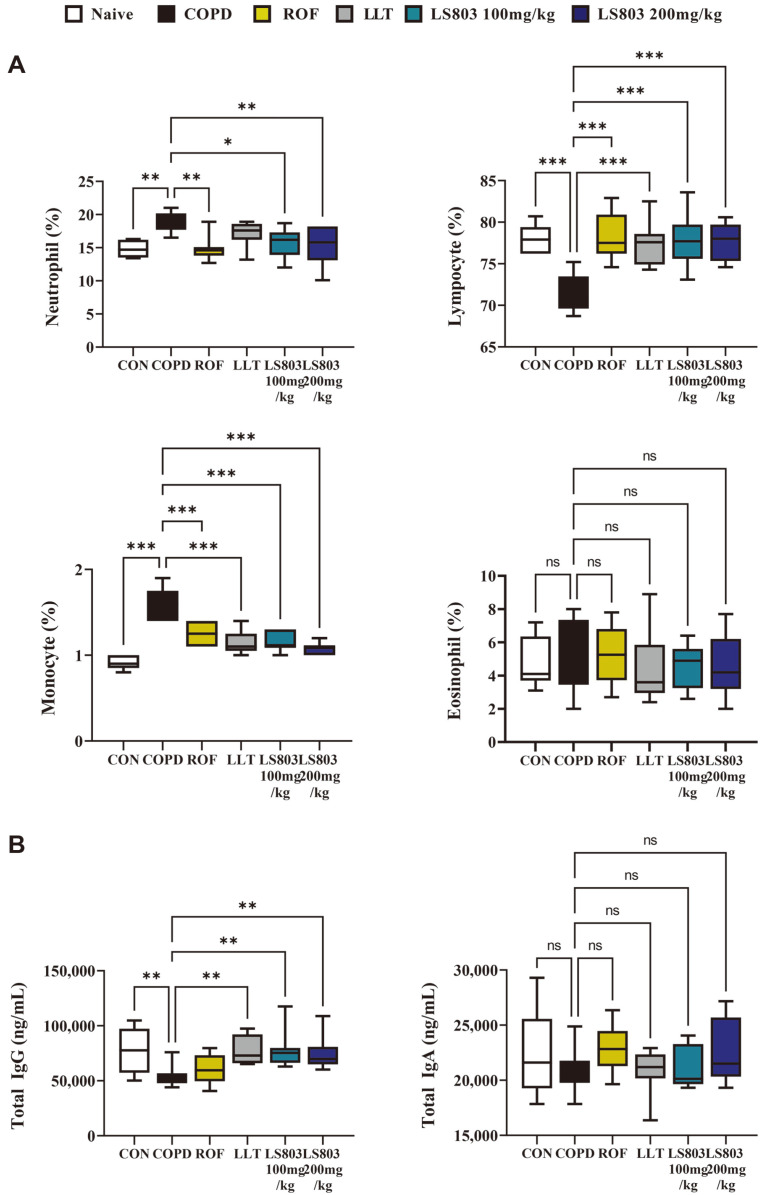
Effect of LS803 on hematological parameters in the COPD model. (**A**) The numbers of neutrophils, lymphocytes, monocytes, and eosinophils in the blood were estimated. (**B**) Immunoglobulin A (IgA) and G (IgG) levels in the serum were measured. Data were analyzed using one-way ANOVA and presented as mean ± SD (*p < 0.05, **p < 0.01, and ***p < 0.001). LLT, bulbs of *Lilium longiflorum* Thunb (the raw material before fermentation); LS803, fermented material.

The levels of IgG were significantly downregulated in the COPD group compared to the naive group. While the levels of IgA were not significantly suppressed, a decreasing trend was observed (Fig. 2B). Hematological analysis revealed that neutrophils and monocytes were significantly more abundant in the COPD group compared to the naive group; the numbers decreased upon administration of LLT and LS803, with LS803 exhibiting a concentration-dependent effect (Fig. 2A). Significantly lower numbers of lymphocytes were observed in the COPD group compared to the naive group and administration of LLT and LS803 tended to recover their levels to that of the naive group. Eosinophils were found at similar levels in all groups, with no significant differences identified (Fig. 2B).

### Effect of LS803 on Airway Inflammation, Alveolar Destruction, and Levels of Cytokines and Chemokines in the Lung Tissue

**Fig. 3:**
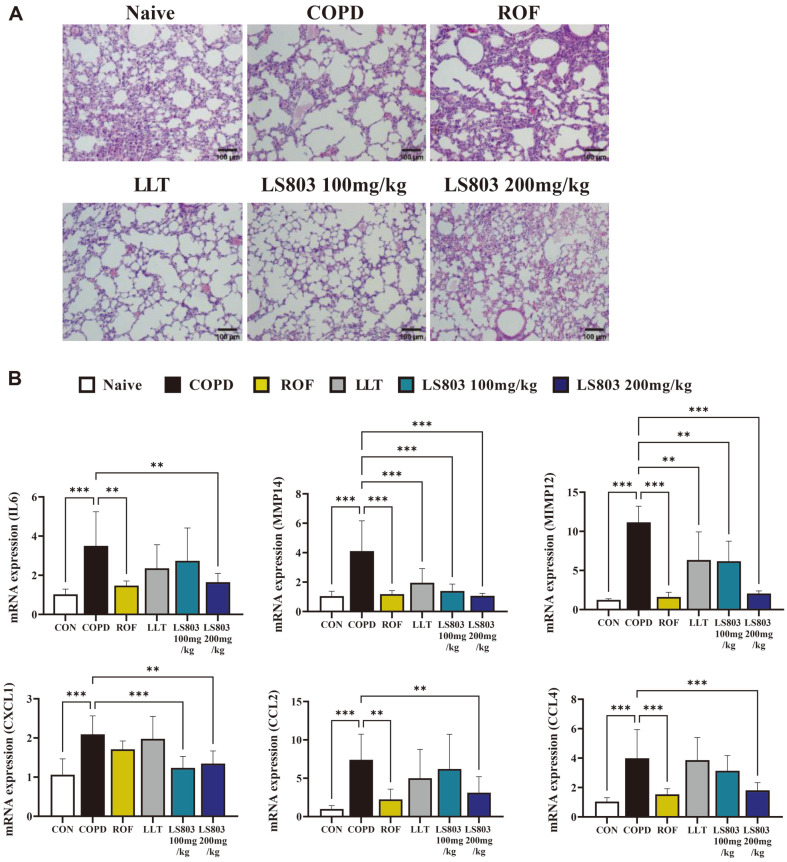
Effect of LS803 on lung destruction and levels of cytokine and chemokines in lung tissues from COPD mice. Lung tissues from each group were collected and used to assess lung destruction and inflammation. (**A**) Lung damage was confirmed using hematoxylin and eosin staining, followed by observation under a microscope (scale bar: 100 μm). (**B**) The mRNA expression levels of inflammation-related markers were measured using quantitative real-time polymerase chain reaction. The data were analyzed using one-way ANOVA. All values have been reported as mean ± SD. (*p < 0.05, **p < 0.01, and ***p < 0.001). LLT, bulbs of *Lilium longiflorum* Thunb (the raw material before fermentation); LS803, fermented material.

Hematoxylin and eosin staining of the lung tissues in each group revealed that the CSE-treated groups showed significant disruption of elastic fibers compared to that in the naive group, with a reduction in the degree of disruption after treatment with LS803 (Fig. 3). Treatment with CSE and PPE significantly induced the production of inflammatory cytokines and chemokines, such as IL-6, MMP-12, MMP-14, CXCL-1, CCL-2, and CCL-4 in the lung tissues, compared to the naive group. Oral administration of LLT and LS803 suppressed the levels of the inflammatory factors, with the administration of LS803 inhibiting most of the inflammatory factors in a concentration-dependent manner (Fig. 3).

### Effect of LS803 on Production of Pro-Inflammatory Cytokines in NCI-H292 Cells

**Fig. 4:**
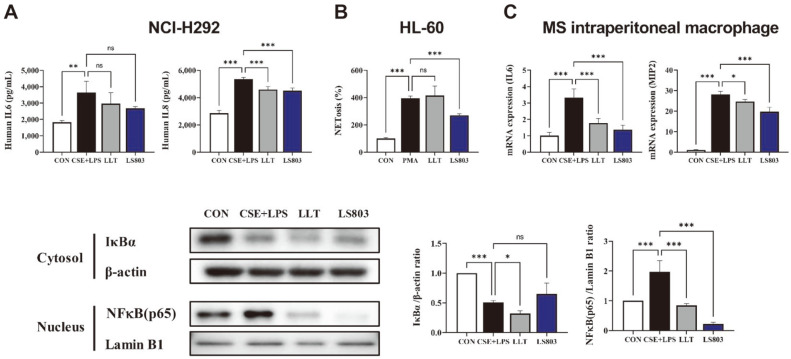
Effect of LS803 on inflammation and NETosis. (**A**) H292 cells were co-treated with 200 μg/ml LLT, 2% CSE and 100 ng/ml LPS for 24 h, after which the levels of human IL-6 and IL-8 in the culture supernatants were measured using ELISA. (**B**) HL-60 cells were co-treated with 200 μg/ml LLT and 100 nM PMA for 3 h, following which the cells were stained with CYTOX Green to assess NETosis. (**C**) Intraperitoneal macrophages were stimulated with CSE and LPS and the expression levels of IL-6 and MIP-2 and NFκB inhibition ability were evaluated. Data were analyzed using one-way ANOVA. All values have been reported as mean ± SD. LLT, bulbs of *Lilium longiflorum* Thunb (the raw material before fermentation); LS803, fermented material.

The effect of LS803 on airway inflammation was confirmed in the human lung epithelial cell line NCI-H292. After stimulation of the cells with 2% CSE and LPS, a significant increase in the levels of both IL-6 and IL-8 was observed in the culture supernatant of the stimulated cells. Treatment with LTT or LS803 led to an attenuation in the production of both IL-6 and IL-8 (Fig. 4A).

### Effect of LS803 on NETosis in HL-60 Cells

As neutrophil recruitment was suppressed in the BALF of COPD-induced mice administered LS803, the human peripheral blood promyeloblast line HL-60 was used as an alternative system for primary neutrophils to evaluate the inhibitory effect of LS803 on neutrophil extracellular traps (NETs). Measuring the NET levels in PMA-stimulated HL-60 cells revealed a significant increase in NETosis compared to the untreated cells. No significant difference was found after LLT treatment; however, NETosis was significantly inhibited after treatment with LS803 (Fig. 4B).

### Effect of LS803 on Pro-Inflammatory Factors and the NFκB Pathway in Peritoneal Macrophages

To confirm the anti-inflammatory effect of LS803, an experiment was conducted using intraperitoneal macrophages. The expression of IL-6 and MIP-2 was significantly increased in macrophages activated by CSE and LPS stimulation; however, when co-treated with LS803, the expression of IL-6 and MIP-2 was significantly decreased (Fig. 4C). The inhibition of NFκB was confirmed in cells treated with LS803 rather than those stimulated with CSE and LPS. These results suggest that LS803 reduces inflammatory mediator production through inhibition of the NFκB pathway (Fig. 4).

## Discussion

Our study identified that LS803, a fermented *Lilium longiflorum* Thunb extract using *Lactobacillus acidophilus*, suppresses CSE- and PPE-induced pulmonary inflammation in a murine COPD model. The major findings of our study are as follows: LS803 inhibited the infiltration of inflammatory cells including macrophages and neutrophils in the BALF of a CSE- and PPE-induced mouse COPD model; in the murine COPD model, LS803 significantly repressed CSE- and PPE-elicited inflammatory gene expression including MIP-1a, IL-12, MDC, and KC in the BALF; LS803 altered CSE- and PPE-induced shifts in the lymphocyte, neutrophil, monocyte, and eosinophil population and function; in the lung tissues of the mouse COPD model, LS803 suppressed CSE- and PPE-induced histological symptoms and inflammatory gene expression including IL-6, MMP-12, MMP-14, CXCL-1, CCL-2, and CCL-4; LS803 repressed CSE- and LPS-induced IL-6 and IL-8 expression in a concentration-dependent manner in the human lung mucoepidermoid carcinoma cell line NCI-H292; LS803 significantly attenuated PMA-induced NETosis in the human promyelocytic leukemia cell line HL-60; and in mouse PMs, LS803 repressed CSE-and LPS-induced IL-6 and IL-8 mRNA expression through the NFκB signaling pathway. Our observations collectively demonstrate that LS803 suppresses the CSE- and PPE-induced COPD-like clinical symptoms and inflammatory gene expression in mouse COPD models.

COPD pathogenesis develops through multiple risk factors such as cigarette smoking, reactive oxygen species, infectious pathogens, acceleration of cell senescence, and inhaled environmental pollutants including biomass fuel, particulate matter, and fumes [27-29]. Recent emerging strategies for understanding COPD etiology suggest that limited exposure to microbes and nutrient deficiency, or inadequate use of antibiotics during pregnancy, may be reprogramming COPD at the fetal stage [30-32]. Because COPD pathogenesis is complex and heterogeneous, studies using genetics and omics are emerging to provide insight into the prevention, diagnosis, and treatment of COPD. A recent genome-wide association study identified 279 variants associated with the heritability of lung function, such as forced expiratory volume in 1second, forced vital capacity, and peak expiratory flow [33]. In particular, SNP rs17486278 at *CHRNA5* and SNP rs11667314 near *CYP2A6* were related to both cigarette smoking and impaired lung function [33]. Another study has shown that rare variants in serpin peptidase inhibitor clade A member 1, which is associated with a1-antitrypsin deficiency, affect lung function, emphysema, and a1-antitrypsin concentration in heavy smokers [34]. Cigarette smoke is enriched with high concentrations of multiple oxidants and increases pro-inflammatory gene expression, including TNF-α, IL-1bβ, IL-6, IL-8, and granulocyte-macrophage colony-stimulating factor, which leads to excessive inflammation and impaired function in the lung and peripheral airways [35, 36]. Furthermore, cigarette smoke is associated with the activation, chemotaxis, and migration of myeloid cells such as monocytes, macrophages, and neutrophils [35]. Macrophages and neutrophils contribute to the initiation and progression of COPD pathogenesis by increasing chronic inflammation, development of emphysema, and airway epithelium remodeling, which are mediated by increased expression of MMPs, neutrophil elastase, myeloperoxidase, and various pro-inflammatory cytokines and chemokines [14, 37]. In addition, COPD-associated alveolar macrophages may contribute to excessive inflammation by defective phagocytosis and apoptosis activity, which are related to mitochondrial dysfunction and altered macrophage metabolism [38]. Cigarette smoke-induced altered adaptive immune response is known to be related to airway epithelium remodeling and obstruction. A study of COPD patients who smoke revealed decreased secretory IgA (sIgA) levels; however, increased airway thickness and excessive infiltration of inflammatory cells in the lungs was observed [39]. Furthermore, cigarette smoking-related deficient sIgA leads to epithelial NFκB activation and increases bacterial invasion and infiltration of inflammatory cells, such as macrophages and neutrophils, through remodeling of the airway epithelium in COPD patients [39]. Our current observations identified that LS803 suppresses CSE- and PPE-induced inflammatory gene expression and histological symptoms such as epithelial cell hyperplasia, elastic fiber disruption, and bronchiole thickness increase, which are mediated by infiltrated lymphocytes, macrophages, and neutrophils in the lung tissues of the COPD mouse. Furthermore, LS803 recovered the CSE- and PPE-induced reduction in IgA and IgG production in the mouse COPD model. In this context, defining the transcriptional or molecular regulation of both innate and adaptive inflammatory cell-mediated inflammation and immune responses may contribute to alternative therapeutic approaches to COPD and many other human ailments including metabolic syndrome, arthritis, and atherosclerosis. Cigarette smoke-induced oxidative stress and inflammation were associated with activated NFκB signaling pathways in the macrophages and epithelial cells of a COPD patient [40]. Consistent with these results, our western blot analyses revealed that CSE and LPS stimulation leads to degradation of IkBa in the cytosol and translocation of NFκB p65 into the nucleus in murine macrophages; however, LS803 significantly inhibited NFκB p65 translocation while reducing IkBa degradation. These observations demonstrate that inhibition of the inflammatory gene expression mediated by NFκB signaling pathways may ameliorate inflammation in the human pulmonary system following CSE exposure.

Inhaled corticosteroids (ICS) have been widely used to treat moderate-to-severe COPD despite their efficacy not being fully proven. Previous clinical studies have revealed that inhaled fluticasone propionate showed no significant clinical benefit on lung function and protease activity and production in the sputum of COPD patients after four weeks of treatment [41]. These observations highlight that more efficacious pharmacotherapies for COPD without harmful side effects, including those with anti-inflammatory, anti-protease, and lung function-enhancing effects, are needed as much as combination therapies of ICS with either long-acting b2 agonists or long-acting muscarinic antagonists. Our current study revealed that LS803 represses lung inflammation, expression of elastolytic enzymes, and leukocyte infiltration in CSE- and PPE-elicited COPD models. This suggests that LS803 may provide beneficial clinical effects for the prevention and treatment of chronic inflammatory diseases including but not limited to COPD, rheumatoid arthritis, ulcerative colitis, and Crohn’s disease.
